# Antibiotic treatment of bacterial vaginosis to prevent preterm delivery: Systematic review and individual participant data meta-analysis

**DOI:** 10.1111/ppe.12947

**Published:** 2023-01-18

**Authors:** Mark A. Klebanoff, Ewoud Schuit, Ronald F. Lamont, Per-Göran Larsson, Hein J. Odendaal, Austin Ugwumadu, Herbert Kiss, Ljubomir Petricevic, William W. Andrews, Matthew K. Hoffman, Andrew Shennan, Paul T. Seed, Robert L. Goldenberg, Lynda M. Emel, Vinay Bhandaru, Steven Weiner, Michael D. Larsen

**Affiliations:** 1 Center for Perinatal Research, The Research Institute at Nationwide Children’s Hospital, Columbus, Ohio, USA; 2 Departments of Pediatrics and Obstetrics and Gynecology, and Division of Epidemiology, The Ohio State University, Columbus, Ohio, USA; 3 Julius Center for Health Sciences and Primary Care, and Cochrane Netherlands, both at University Medical Center Utrecht, Utrecht University, Utrecht, The Netherlands; 4 Division of Surgery, University College London, Northwick Park Institute for Medical Research Campus, London, UK; 5 Odense University Hospital, Department of Gynecology and Obstetrics, University of Southern Denmark, Institute of Clinical Research, Research Unit of Gynecology and Obstetrics, Odense, Denmark; 6 Department of Obstetrics and Gynaecology, Skaraborg Hospital, Skövde, Sweden; 7 Department of Clinical and Experimental Medicine (IKE), Linköping University, Linköping, Sweden; 8 Department of Obstetrics and Gynaecology, Stellenbosch University, Cape Town, South Africa; 9 Department of Obstetrics and Gynecology, St. George’s Hospital, University of London, London, UK; 10 Department of Obstetrics and Gynaecology, Medical University of Vienna, Wien, Austria; 11 Department of Obstetrics and Gynecology, University of Alabama at Birmingham, Birmingham, Alabama, USA; 12 Department of Obstetrics and Gynecology, Christiana Health Services, Newark, Delaware, USA; 13 Department of Women and Children’s Health, School of Life Course Sciences, FoLSM, King’s College, London, UK; 14 Division of Women’s Health, King’s College, London, UK; 15 Department of Obstetrics and Gynecology, Columbia University, New York City, New York, USA; 16 Biostatistics, Bioinformatics, and Epidemiology/VIDD, Fred Hutchinson Cancer Center Seattle, Seattle, Washington, USA; 17 The Biostatistics Center, Milken School of Public Health, The George Washington University, Washington, District of Columbia, USA; 18 Department of Mathematics and Statistics, St. Michael’s College, Colchester, Vermont, USA

**Keywords:** bacterial vaginosis, clindamycin, individual participant data, meta-analysis, metronidazole, preterm delivery, systematic review

## Abstract

**Background:**

Bacterial vaginosis (BV) increases preterm delivery (PTD) risk, but treatment trials showed mixed results in preventing PTD.

**Objectives:**

Determine, using individual participant data (IPD), whether BV treatment during pregnancy reduced PTD or prolonged time-to-delivery.

**Data Sources:**

Cochrane Systematic Review (2013), MEDLINE, EMBASE, journal searches, and searches (January 2013–September 2022) (“bacterial vaginosis AND pregnancy”) of (i) clinicaltrials.gov; (ii) Cochrane Central Register of Controlled Trials; (iii) World Health Organization International Clinical Trials Registry Platform Portal; and (iv) Web of Science (“bacterial vaginosis”).

**Study Selection and Data Extraction:**

Studies randomising asymptomatic pregnant individuals with BV to antibiotics or control, measuring delivery gestation. Extraction was from original data files. Bias risk was assessed using the Cochrane tool. Analysis used “one-step” logistic and Cox random effect models, adjusting gestation at randomisation and PTD history; heterogeneity by *I*^2^. Subgroup analysis tested interactions with treatment. In sensitivity analyses, studies not providing IPD were incorporated by “multiple random-donor hot-deck” imputation, using IPD studies as donors.

**Results:**

There were 121 references (96 studies) with 23 eligible trials (11,979 participants); 13 studies (6915 participants) provided IPD; 12 (6115) were incorporated. Results from 9 (4887 participants) not providing IPD were imputed. Odds ratios for PTD for metronidazole and clindamycin versus placebo were 1.00 (95% CI 0.84, 1.17), *I*^2^ = 62%, and 0.59 (95% CI 0.42, 0.82), *I*^2^ = 0 before; and 0.95 (95% CI 0.81, 1.11), *I*^2^ = 59%, and 0.90 (95% CI: 0.72, 1.12), *I*^2^ = 0, after imputation. Time-to-delivery did not differ from null with either treatment. Including imputed IPD, there was no evidence that either drug was more effective when administered earlier, or among those with a PTD history.

**Conclusions:**

Clindamycin, but not metronidazole, was beneficial in studies providing IPD, but after imputing data from missing IPD studies, treatment of BV during pregnancy did not reduce PTD, nor prolong pregnancy, in any subgroup or when started earlier in gestation.

## Abstract (summary)

Tweetable AbstractIn a meta-analysis of bacterial vaginosis treatment in pregnancy and preterm delivery (PTD), we used individual participant data (IPD), imputing nonresponding studies. With only IPD clindamycin showed benefit to reduce PTD. Incorporating imputed studies we found no benefit of treatment, cumulatively or in any subgroup.

## 1 | BACKGROUND

Globally, preterm delivery (PTD) is the leading cause of mortality among children <5 years.^1^ Intrauterine infection is a leading cause of PTD,^2^ particularly at early gestations.^3,4^ Most intrauterine infection is due to ascending colonisation from the lower genital tract to the uterus before or during pregnancy,^5^ and cultures of the chorioamniotic membranes of preterm infants have implicated bacterial vaginosis (BV) associated bacteria.^6^ BV has been observed in 29% of reproductive-age individuals in the United States,^7^ and occurs when the usual *Lactobacillus*-dominant vaginal microbiota is replaced by an overgrowth of a diverse, predominantly anaerobic microbiota.^8,9^ Overall, asymptomatic pregnant individuals with BV are at >2-fold increased risk of PTD compared with those without BV,^10^ >5-fold risk when BV is diagnosed before 20 completed weeks’ gestation,^11^ and >7-fold increased risk when BV is diagnosed before 16 completed weeks’ gestation,^12^ raising the question of whether treatment of BV early in pregnancy might be effective at preventing PTD.

There have been numerous clinical trials to treat asymptomatic BV with antibiotics to prevent PTD,^13–35^ that generated many systematic reviews.^36–52^ Four reviews concluded that treatment of BV with antibiotics does not reduce PTB among low-risk, but may be effective in high-risk individuals.^37,38,40,42^ One concluded that treatment is ineffective in high-risk but may be effective in low-risk individuals.^50^ One concluded that treatment was ineffective in low or average risk but could not reach a conclusion regarding high-risk individuals.^52^ Two concluded that treatment is ineffective in general but may be effective when administered earlier in pregnancy,^45^ particularly treatment with clindamycin,^41^ and six reported that treatment is ineffective to prevent PTB, overall or in any subgroup.^36,43,44,46–48^ All previous reviews were based only on aggregate, published data from each trial, rather than pooled, individual participant data (IPD). In contrast to aggregate data, IPD meta-analysis combines raw data from each trial while preserving the clustering of participants within trials.^53,54^ IPD meta-analysis has more power than conventional meta-analyses to detect subgroup differences,^55,56^ and permits a common definition of subgroups and outcomes. Since prolongation of pregnancy, even if not sufficient to demonstrate a reduction in total PTD, is clinically relevant, IPD including exact gestation at delivery will allow determination of time from randomisation to delivery. This investigation is an IPD meta-analysis of randomised clinical trials of antibiotic treatment of asymptomatic BV during pregnancy to prevent PTD, incorporating studies where IPD could not be obtained. We hypothesised that compared to placebo, or no treatment, antibiotics will be more effective: (i) in preventing PTD the earlier in pregnancy they are administered; (ii) in preventing PTD in individuals with a previous PTD; and (iii) will result in a greater randomisation-to-delivery interval. A secondary hypothesis was that the effect of treatment differed between metronidazole and clindamycin.

## 2 | METHODS

### 2.1 | Search Strategy

The review protocol was registered with PROSPERO (CRD4201 5020304) on 6 May 2015. Criteria for inclusion were (i) enrolled pregnant individuals with BV, regardless of how diagnosed; (ii) participants randomised to an antibiotic or placebo/no treatment/presumed inactive treatment; (iii) measured gestational duration at the end of pregnancy; and (iv) provided original study data. Studies were excluded if they recruited women following an episode of spontaneous preterm labour (SPTL) or preterm prelabour rupture of the membranes (PPROM). The search began with the 2013 Cochrane systematic review of treating BV during pregnancy.^36^

We supplemented the 2013 Cochrane Review by searching the Cochrane Central Register of Controlled Trials and clinicaltrials.gov using the term “bacterial vaginosis” on 8 January 2013, and bacterial vaginosis AND pregnancy, on 26 August 2014, 19 May 2016, and 18 July 2016. The WHO ICTRP portal was searched with “bacterial vaginosis” AND “pregnancy” on 28 July 2016. A Web of Science Core Collection: Citation Indices search was conducted weekly to 31 December 2016 using the term “topic = vaginosis, bacterial” to identify eligible publications. Study summaries were reviewed by M.K. and, for studies identified in clinicaltrials.gov and the ICTRP portal, S.W., to determine eligibility for inclusion. The reference lists of all eligible studies were searched. A search of the Cochrane Central Register was conducted by M.K. (terms “bacterial vaginosis” AND “pregnancy”) on 23 August 2017. In 2020, we cross-referenced our identified studies against the summary of screening for BV to prevent PTD conducted by the U.S. Preventive Services Task Force,^57^ which searched the literature through December 2019, and we found no new eligible studies. On 29 September 2022 we searched clinicaltrials.gov (“bacterial vaginosis AND pregnancy”), the WHO ICTRP portal (“bacterial vaginosis”), and the Cochrane Central Register of Controlled Trials (“bacterial vaginosis”) for 2020–2022 to capture trials published after the Task Force review, and we continued our weekly Web of Science search. No additional eligible studies were identified.

### 2.2 | Study selection

We attempted to contact the first or corresponding author of eligible studies. If that proved impossible, other authors were contacted. For ongoing studies, we contacted the study representative. Participating investigators were asked to forward anonymised data in any format, along with any available documentation, to the George Washington University Biostatistics Center. Once data were received, the variables and published articles were reviewed to confirm that the number and characteristics of participants as described in the article could be replicated. Any discrepancies were pursued with the study investigator. Common definitions of variables across studies were developed, and the variables were edited and cleaned as needed. In participants with intermediate (4–6) and BV (7–10) Nugent Gram stain scores, only those with scores of 7–10 were included. If they could not be differentiated, all Nugent scores 4–10 were included. Regardless of how the original study was analysed, we included all randomised participants with outcome data available, following intention-to-treat.

**FIGURE 1:**
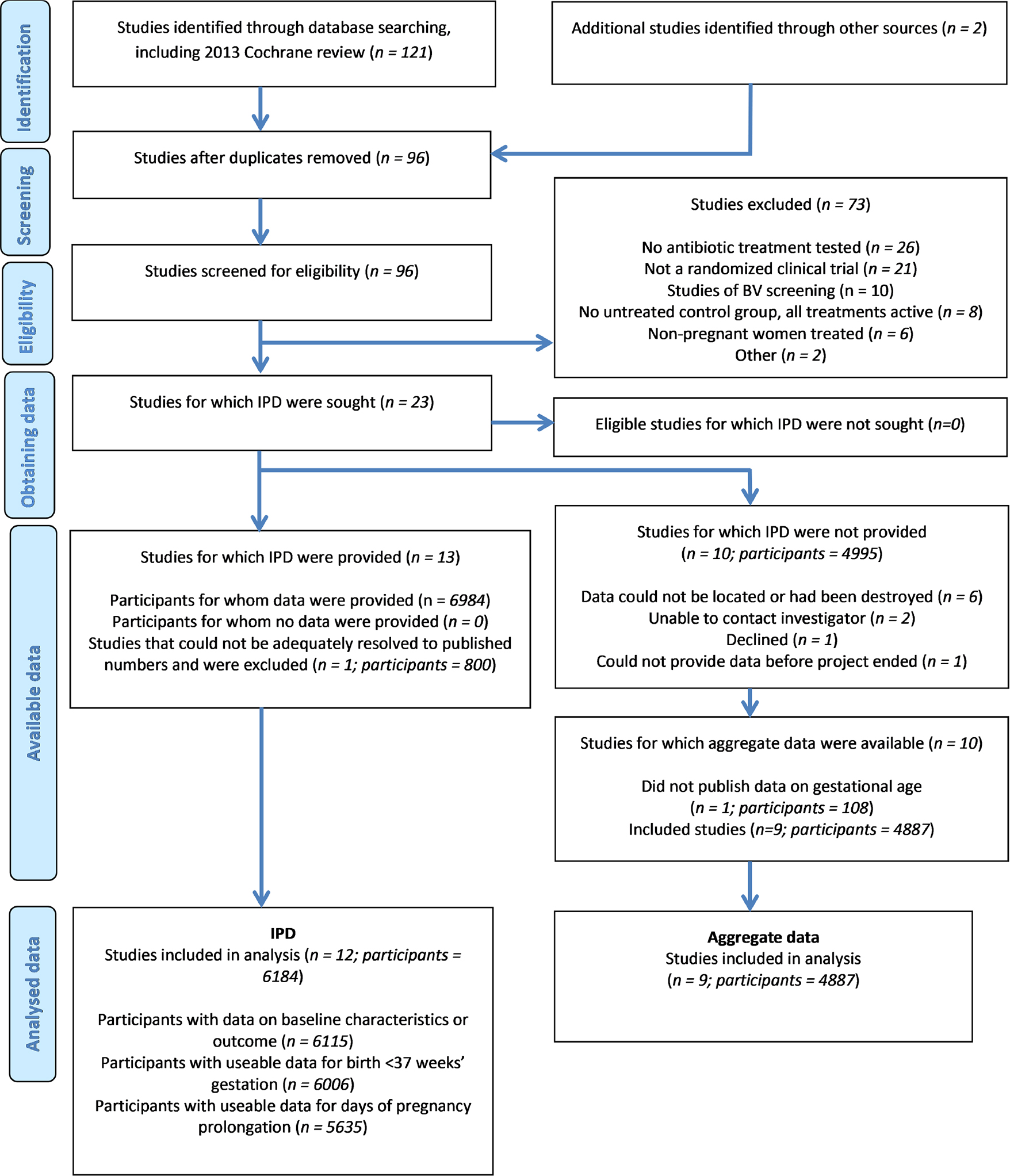
Flow of study identification

Study selection is summarised in Figure 1. Our search identified 121 entries from 96 unique studies (one publication^58^ was a subset of another^22^), which included two studies identified from references of searched papers, or from investigator knowledge.^18,34^ Neither of these two studies was identified by the 2013 Cochrane Review. Seventy-three of the 96 studies (including three from the Cochrane Review) were ineligible for the reasons noted in Figure 1. The Cochrane Review also identified but had to exclude one study that did not publish aggregate data on the subgroup of enrolled individuals who had BV. However, we were able to gain access to IPD from this study for inclusion.^16^ Risk of bias for each study was evaluated using the Cochrane Collaboration tool.^59^ Evaluation of all eligible studies was done independently by M.K. and either E.S. or S.W., with discrepancies resolved by consensus, and was shared with the original investigators where necessary. Criteria were judged as low, high, or unclear risk of bias. All studies were included in the analysis, regardless of the risk of bias, and studies were not weighted by quality score.^59^

### 2.3 | Outcomes

The primary outcomes were PTD and continuous GA at delivery. All but three of the studies providing IPD^16,18,60^ determined GA by sonography, and a fourth used sonography for participants randomised before 24 completed weeks’ gestation.^29^ Secondary outcomes were birth before 34 and 32 completed weeks’ gestation, perinatal death, SPTL, PPROM, clinical chorioamnionitis, and admission to the neonatal intensive care unit.

### 2.4 | Statistical analysis

Data analysis followed a “one-step” approach. Logistic regression, with a random effect for the study to capture additional study heterogeneity, was used to model the probability of PTD. Time to delivery was assessed by survival analysis, using a Cox proportional hazards model with a random effect for study, with GA at birth capped at 37 completed weeks’ gestation. We also fitted adjusted models with covariates of GA at randomisation (continuous), and history of PTD, which were our pre-specified subgroup analyses. We used two definitions of PTD history: (i) a 2-level variable (yes or no) and (ii) a 3-level variable (yes, no, and no previous births) but the results were similar with either definition. In both logistic and Cox models, we tested the pre-specified hypotheses that treatment was more effective when administered earlier in pregnancy and was more effective in participants with a previous PTD, by including interaction terms between treatment and GA at randomisation, and between treatment and obstetric history, respectively. We also tested whether metronidazole and clindamycin differed in their ability to prevent PTD (a pre-specified secondary hypothesis) by including interaction with the type of antibiotic. When subgroup interactions were statistically significant, stratified analyses were done.

We assessed between-study heterogeneity of results for the odds ratio (OR) for birth before 37 completed weeks’ gestation and hazard ratio (HR) for delivery by the *I*^2^ statistic.^61^ Heterogeneity (*I*^2^ values) of 25%, 50%, and 75% represented low, moderate, and high heterogeneity, respectively. Negative values are conventionally reported as 0.^61^ Since some studies excluded twin pregnancies, the results are presented as all pregnancies, and singleton pregnancies alone.

### 2.5 | Missing data and sensitivity analysis

To address the impact of missing IPD studies, we performed an additional analysis in which their results were imputed. Imputation was preferred to a 2-stage approach to combine IPD and aggregate data to allow incorporation of trials that did not report results in a way that would contribute to our meta-analysis. Random hot-deck donor multiple imputations were done separately for studies using metronidazole and clindamycin.

In brief, for each study not providing IPD the cell totals of the treatment x PTD table were known, although the individual-level variable values, including the exact gestational ages at randomisation and delivery, were not. Data from studies providing IPD were used as “donors” for the missing studies. Two separate donor pools were established (for clindamycin and metronidazole) utilising the IPD studies. The exclusion and inclusion criteria from each missing study were applied to the pool, which was then randomly sampled with replacement and unequal probability according to treatment and outcome to duplicate the 2 × 2 table from each missing study. Each study was created randomly five times to reflect uncertainty. The results of five separate analyses were combined using rules for multiple imputations for missing data.^62^ Details of the imputation procedure are included in the supplemental material. Finally, we determined the sensitivity of the results to the inclusion of any single study by sequentially excluding one study at a time to determine the effect on the total results.

### 2.6 | Ethics approval

This study was approved as part of the George Washington University Biostatistics Center-wide Institutional Review Board application. The study protocol was registered with PROSPERO (CRD42015020304) on 6 May 2015, and edited on 4 November 2017 and 19 January 2017.

## 3 | RESULTS

### 3.1 | Description of Studies

IPDs were requested from all 23 eligible trials. Investigators from 13 studies, representing 6915 participants, provided data. We obtained IPD from seven studies that used metronidazole^13,16,19,29,30,34,35^ and six that used either oral or intravaginal clindamycin.^18,20,23–25,32^ We were unable to obtain IPD from three metronidazole^26–28^ and six clindamycin studies^15,17,21,22,31,33^ (Figure 1). IPD from one study^23^ provided GA at birth as a categorical variable (before 33, 33–36, and at least 37 completed weeks’ gestation). Tables S1 and S2 provide characteristics of eligible studies with and without IPD, respectively. We were unable to reconcile the data provided from one study^18^ to its published results. This study recruited individuals with BV or candidiasis and did not publish results separately for those with BV, and they could not be identified from the data provided. Accordingly, this study was excluded from the remainder of the project, leaving 12 IPD studies totaling 6115 participants, 6006 of whom could be analysed. The number of participants with BV in the included IPD studies ranged from 13 to 1944. GA at randomisation ranged from 10 to 27 weeks. One study not providing IPD reported only that the duration of gestation did not differ significantly between the treated and control groups,^14^ so was excluded from the project. The sample size among the remaining nine studies not providing IPD was 4778, (range 22–2869); five studies used intravaginal clindamycin,^15,17,21,22,33^ three oral metronidazole,^26–28^ and one oral clindamycin.^31^ The GA at randomisation ranged from 10 to 34 weeks. The large majority of studies with and without IPD diagnosed BV by Nugent score, though some also required an elevated pH or other Amsel criteria.^63^

The risk of bias determinations for studies with and without IPD are included in Tables S3 and S4, respectively. Most studies that provided IPD scored well in the determination. By design, two studies could not be completely blinded. These studies used a modified Zelen design^64^ in which all participants consented to be screened for BV, but consent for treatment was only sought from those randomised to screening, if they had BV.^23,25^ In the context of the Zelen design, we did not consider incomplete blinding to be a serious limitation. One study^29^ was deemed to be at high risk of bias for certain characteristics. The control treatment was visibly different from the active treatment, leading to the post-randomisation exclusion of participants who deliberately received active treatment while assigned to control. This was not noted in the study publication but was revealed by the investigator (who previously had been unaware of this) when study files were reconciled to the published paper. The study was retained in the IPD meta-analysis, as our pre-planned sensitivity analysis would evaluate whether any single study had a major impact on the overall results.

Although the quality of studies that did not provide IPD was more variable, the larger studies were, in general, at low risk of bias. A common shortcoming, regardless of the provision of IPD, was our inability to obtain original protocols. Accordingly, we could not determine selective reporting of outcomes, nor whether the original primary outcome agreed with that in the publication. These studies were deemed of unclear risk for this bias.

Table 1 presents the selected baseline characteristics of participants in the IPD studies. The treated and control groups were well balanced, although not all studies provided data on all the characteristics in the table.

### 3.2 | Effect of treatment of BV on pregnancy outcome in studies providing IPD

The effects of BV treatment on the OR for PTD and HR for GA at birth for each individual study that provided IPD are presented in Tables S5 and S6, respectively. The overall effect of BV treatment on pregnancy duration is presented in Table 2. Regardless of the inclusion of twins, treatment did not reduce the occurrence of PTD. The ORs for birth before 34 and before 32 completed weeks’ gestation were of similar magnitude. One study could not be included in the time-to-event analysis because GA at birth was provided as a 3-level categorical variable.^23^ In a time-to-event analysis with the remaining studies, we found no evidence that treatment prolonged pregnancy, regardless of adjustment for GA at randomisation, history of PTD, or inclusion of twins. None of the p-values for interaction were statistically significant, implying that the effect of treatment did not differ by GA at randomisation or history of PTD.

However, the treatment effect for both PTD and time-to-delivery differed by whether the active drug was clindamycin or metronidazole. Because of the interaction, we present all further results separately for studies that used metronidazole (Table 3) and clindamycin (Table 4). As shown in Table 3, metronidazole did not have a clinically relevant effect on any study outcome. Table 4 shows that clindamycin treatment reduced PTD regardless of the exclusion of twins. The ORs for birth at earlier GAs were of similar magnitude. Clindamycin treatment showed an increased time-to-delivery.

In evaluating whether the treatment effect differed by GA at randomisation, few participants were randomised to metronidazole before 16 weeks, and few to clindamycin after 22 weeks. We found no evidence that the effect of metronidazole on any outcome differed by GA at randomisation or history of PTD (Table 3 and Figures S1 and S2, top row). However, in the IPD studies, the effect of clindamycin on time-to-delivery differed by gestation at randomisation (Table 4 and Figure S3, top row). Clindamycin may have been more effective when administered from 20 to 22 weeks than when administered earlier. The effect of clindamycin did not differ between participants with or without a history of PTD (Table 4 and Figure S4, top row). Pre-specified secondary outcomes not already presented are in Table S7. Not every study collected data on every secondary outcome. We found no evidence that treatment improved any secondary outcome.

### 3.3 | Effect of including studies from which IPD were not obtained

We imputed IPD from those studies from which only aggregate, published results were available. As shown in Table 5 and Figures S1 and S2 (second row), the imputation of IPD did not have a meaningful impact on the overall or subgroup analyses results for metronidazole. However, Table 6 shows that results for clindamycin were substantially changed after the imputation of IPD. Following the imputation of missing IPD, the OR for PTD and the time-to-delivery moved toward unity. After the imputation of IPD, the interactions between treatment assignment and either gestational age at randomisation or history of PTD were no longer statistically significant in any model (Table 6 and Figures S3 and S4, second row).

The effect of individual studies on the imputed results is presented in Table S8 for ORs for PTD and S9 for HRs for GA at birth. Neither metronidazole nor clindamycin results for either PTD or GA at birth was substantially changed by the exclusion of any one study. Although PREMEVA^31^ was the largest clindamycin study and its results were, in contrast to existing data, null,^65^ it was not solely responsible for the change in effect when all missing studies were imputed. Imputation of IPD from all clindamycin studies except PREMEVA resulted in effects on PTD and gestational length that were still reduced (Tables S8 and S9).

## 4 | COMMENT

### 4.1 | Principal Findings

This systematic review and IPD meta-analysis, which imputed data from studies not providing IPD, found no evidence among IPD studies that treatment of BV without regard to antibiotic prevented PTD, nor prolonged pregnancy, but found that metronidazole and clindamycin had different effects. There was no evidence that metronidazole treatment improved any measure of pregnancy duration, in aggregate or among any subgroup; nor did imputation of missing studies^26–28^ alter this conclusion, although there was moderate between-study heterogeneity. However, the IPD studies employing clindamycin reported reduced occurrence of PTD with some evidence for increased time-to-delivery, but after imputation of clindamycin studies,^15,17,21,22,31,33^ the protective effect of clindamycin on PTD was reduced, and there was no evidence that treatment effectiveness differed by gestation at randomisation. There was little statistical heterogeneity between clindamycin studies, either before or after imputation.

### 4.2 | Strengths of the study

A weakness of aggregate data meta-analysis is that published outcomes may be defined differently between studies. In addition, it is impossible to study either time-to-delivery or GA at randomisation as continuous variables based on published aggregate data. Furthermore, clinical trials may not have reported results of all subgroups of interest, and when they did report such results, they may have used different subgroup definitions. IPD meta-analysis avoids these limitations because individual-level data allowed us to derive consistent definitions of subgroups and outcomes, study the role of GA at randomisation on treatment effectiveness, and conduct an analysis of GA at delivery as a continuous variable.

### 4.3 | Limitations of the data

Foremost among the limitations is that either we could not obtain IPD from all studies, or we could not reconcile IPD with published results. We addressed missing IPD by utilising the IPD we had as donors for the “missing” participants. Although our imputed studies matched the aggregate results and covariate distributions as stated in the relevant study publications, our imputation might not be accurate. Nevertheless, we believe it is preferable to impute these studies than to ignore them. Our imputation was designed to match the marginal tables of the trials not providing IPD, so results involving study-level characteristics, such as choice of antibiotic, should not differ from aggregate data meta-analysis. However results involving individual-level factors, such as an interaction between treatment and GA at randomisation or the effect of treatment on time-to-delivery, maybe in error if our imputation was incorrect. Second, our search strategy, which began with the 2013 Cochrane review, has been criticised for not considering the timing of treatment, specifics of the BV diagnosis, inclusion based only on the history of PTB, and whether participants were re-screened and if positively re-treated,^65^ and may have missed eligible studies. However, the US Preventive Services Taskforce conducted a search through December, 2019^57^ and did not find any trials of which we were unaware. The 23 studies we identified randomised over 11,000 participants. It seems unlikely that a study sufficiently large to have changed our results was missed. Future IPD meta-analyses could be improved by obtaining IPD on the studies we were unable to access, particularly the large PREMEVA study,^31^ although a common reason we could not access non-PREMEVA IPD was that the relevant studies were old and the data no longer accessible.

### 4.4 | Interpretation

Intrauterine infection has been more strongly associated with spontaneous than with indicated PTD,^5^ and treatment of BV may be more effective to prevent spontaneous than indicated PTD. Unfortunately the presenting characteristics of the PTD were not consistently included in the data files, and we could not address this. When they could be identified, we studied only participants with Nugent scores of 7–10, but if not, all participants were included, regardless of the Nugent score. However, even within this score range, those with higher scores are at increased risk of PTD birth compared to those with lower scores,^32,66^ and oral treatment may be more beneficial in individuals with higher scores.^32^ Very few data files included Nugent scores, so we could not investigate this. Finally, the occurrence of

BV remission in the control group ranged from 14%^28^ to 70%,^17^ with typical “spontaneous remission” rates of approximately 30–40%. We did not evaluate whether studies that systematically re-screened and re-treated persistently positive participants produced more favourable results than studies that did not, though there is evidence to support this.^67^ One study^22^ noted that those who, following intervention, had persistent BV compared to those that were cured, had a ~3-fold increased risk of late miscarriage and PTD. Similarly, those cured but with recurrent BV compared with no recurrence had approximately 10-fold increased risk of late miscarriage or PTB.

The Nugent Gram stain^68^ is considered the gold standard for diagnosing BV in research studies. Several of the trials, however, diagnosed BV only by Amsel criteria,^15,27,28^ or by the Spiegel Gram stain score with or without Amsel clinical criteria.^19,22,26,29^ Several of the Amsel criteria are subjective, and the Nugent score manifests better inter-observer agreement than the Spiegel score.^68^ If BV was misdiagnosed, then antibiotics active against BV would not be expected to prevent PTD. Several molecular-based diagnostic tests for BV are licensed for use in the United States or Europe.^69^ These multiplex PCR tests have favourable sensitivity, specificity, and positive and negative predictive value,^70^ and because they require less observer judgement should be considered to replace microscopy diagnosis of BV in future research. In the past decade, DNA-based methods of characterising the vaginal microbiota have emerged^9^ and are providing new information^71^ although they have not yet provided consistent evidence on the role of the vaginal microbiota and PTD.^72–75^ While the existing evidence is sparse and contradictory, ultimately these methods may provide insights regarding those individuals who might benefit from antimicrobial treatment during pregnancy.^76^

We identified 14 systematic reviews or aggregate-data meta-analyses to treat BV to prevent PTD, results of which have been highly inconsistent.^36–38,40–48 ,50,52^ Differences in inclusion and exclusion criteria, including failure to separate metronidazole and clindamycin studies and failure to consider the timing of treatment or differences in study entry criteria,^77^ as well as true differences over time as the completed trials accumulated, may account for the divergent results. Had we based our analysis solely on studies providing IPD, we would have reported that treatment with clindamycin was effective at preventing PTD. When we imputed study results for which we had only aggregate data, our results agreed with the 2013 Cochrane review that treatment was ineffective at preventing PTD regardless of the individual’s risk status or when treatment was administered.^36^ Excluding the imputed results of the large PREMEVA study,^31^ or any other single study, did not change this conclusion. Although PREMEVA diagnosed BV by Nugent score, questions have been raised whether independent review of only 1% of Gram stain slides was sufficient to ensure a reliable diagnosis, given that BV was assessed in 149 different laboratories, few of which had previous experience with the score.^77^ An investigator from PREMEVA has acknowledged concerns over the diagnosis of BV and publication was delayed while molecular confirmation of the diagnosis was to be carried out, albeit the published findings did not note this.^78^

## 5 | CONCLUSIONS

Results among studies providing IPD indicated that clindamycin, but not metronidazole, reduced the risk of PTD among participants with BV. However, the inclusion of imputed IPD from studies not providing IPD found that neither drug reduced PTD. The imputed results are generally consistent with the recommendations of many official bodies including the U.S. Preventive Services Task Force (for pregnant individuals at low risk of PTD),^57^ U.S. Centers for Disease Control and Prevention,^79^ the National Institute for Health and Care Excellence (NICE),^80^ and the Danish Society of Obstetrics and Gynecology,^81^ although the latter has been criticised.^77^ Nevertheless, some uncertainty remains. The PHS Task Force report found insufficient evidence to assess the balance of benefit or harm of screening for BV in individuals at high PTD risk,^57^ the Society of Obstetricians and Gynaecologists of Canada states “Women at increased risk for preterm birth may benefit from routine screening for and treatment of bacterial vaginosis,”^82^ and the CDC documents on the topic occasionally are contradictory.^83^ Finally, while included trials used PTD and prolongation of pregnancy as outcome parameters, these are surrogate markers. We encourage future studies to focus on neonatal outcomes as the main marker for the benefit or harm of any intervention.

## Supplementary Material

supplementary appendix

supplementary figures

supplementary tables

supplementary figure s4

## Figures and Tables

**TABLE 1:** Characteristics of studies providing individual participant data

Characteristic^a^	Treatment	Placebo
Twin pregnancy	0.6%	0.8%
Previous preterm risk		
Nulliparous	47.7%	48.5%
Multiparous, no previous preterm	39.1%	40.5%
Multiparous, previous preterm	12.8%	10.5%
Missing	0.5%	0.5%
Smoking		
Smoking	13.7%	14.3%
Not smoking	48.2%	50.2%
Missing/unknown	38.1%	35.5%
Race		
Black	33.4%	31.3%
Other	6.4%	7.4%
White	14.8%	14.0%
Missing/unknown	45.4%	47.2%
Mean ± SD body mass index, kg/m^2^	25.2 ± 5.6	25.2 ± 5.9
Body mass index missing	25.1%	22.0%
Mother’s age ± SD	24.8 ± 5.7	24.7 ± 5.6
Mean ± SD weeks’ gestation at randomisation	18.7 ± 3.9	18.7 ± 3.9
Median (IQR) weeks’ gestation at randomisation	19 (6)	19 (6)

a Results are based on 3093 treated and 3022 control women, except for BMI (2317 and 2357 women), mother’s age (2902 and 2830 women), and gestational age at randomisation (3087 and 3020 women).

**TABLE 2:** Effect of treatment on measures of pregnancy duration, studies providing Individual Participant Data

Results	Treated (*n*)^a^	Controls (*n*)^a^	Estimate of treatment effect^b^	*p*-value for interaction of treatment and gestation at randomisation	*p*-value for interaction of treatment and preterm history	*p*-value for interaction of treatment and drug (CM vs. MZ)
Birth <37weeks			Odds ratio (95% CI)			
Including twins	3041	2965	0.90 (0.77, 1.04)	.15	.48	.007
Singletons only	3023	2940	0.91 (0.79, 1.06)	.26	.57	.017
Birth <34weeks			Odds ratio (95% CI)			
Including twins	2863	2786	0.86 (0.68, 1.08)	.95	.24	.23
Singletons only	2846	2763	0.87 (0.69, 1.10)	.75	.27	.35
Birth <32weeks			Odds ratio (95% CI)			
Including twins	2864	2786	0.96 (0.74, 1.27)	.97	.05	.24
Singletons only	2847	2763	0.99 (0.75, 1.31)	.76	.07	.41
Time until birth			Hazard ratio (95% CI)			
Including twins	2856	2779	0.99 (0.96, 1.02)	.37	.59	.050
Singletons only	2839	2756	0.99 (0.97, 1.02)	.51	.62	.098

Abbreviations: CM, clindamycin; MZ, metronidazole.

a In all studies, there are 3093 treated and 3022 control subjects. Some studies are missing one or more variables needed for analysis.

b Logistic model with random effect of study for the odds ratio and terms for treatment/control, gestational age at randomisation, and history of preterm birth; comparable Cox model for hazard ratio.

**TABLE 3:** Effect of treatment on measures of pregnancy duration (metronidazole trials providing individual participant data)

Results	Treated (*n*)^a^	Controls (*n*)^a^	Estimate of treatment effect^b^	*p*-value for interaction of treatment and gestation at randomisation	*p*-value for interaction of treatment and prior preterm history
Birth <37weeks			Odds ratio (95% CI)		
Including twins	2022	1950	1.00 (0.84, 1.17)	.99	.45
Singletons only	2013	1943	1.00 (0.85, 1.18)	.93	.47
*I^2^*			62%		
Birth <34weeks			Odds ratio (95% CI)		
Including twins	2022	1952	0.92 (0.71, 1.19)	.41	.42
Singletons only	2013	1945	0.92 (0.71, 1.19)	.42	.42
Birth <32weeks			Odds ratio (95% CI)		
Including twins	2023	1952	1.05 (0.77, 1.43)	.44	.13
Singletons only	2014	1945	1.05 (0.77, 1.43)	.45	.13
Time until birth			Hazard ratio (95% CI)		
Including twins	2021	1948	1.01 (0.98, 1.04)	.83	.40
Singletons only	2012	1941	1.01 (0.98, 1.04)	.88	.40
*I^2^*			16%		

a In all studies, there are 2047 treated and 1979 control subjects. Some are missing one or more variables needed for analysis.

b Logistic model with random effect of study for the odds ratio and terms for treatment, gestational age at randomisation, and history of preterm birth; comparable Cox model hazard ratio.

**TABLE 4:** Effect of treatment on measures of pregnancy duration (clindamycin trials providing individual participant data)

Results	Treated (*n*)^a^	Controls (*n*)^a^	Estimate of treatment effect^b^	*p*-value for interaction of treatment and gestation at randomisation	*p*-value for interaction of treatment and preterm history
Birth <37weeks			Odds ratio (95% CI)		
Including twins	1019	1015	0.59 (0.42, 0.82)	.37	.68
Singletons only	1010	997	0.62 (0.43, 0.88)	.25	.60
*I^2^*			0^c^		
Birth <34weeks			Odds ratio (95% CI)		
Including twins	841	834	0.64 (0.38, 1.07)	.41	.41
Singletons only	833	818	0.69 (0.40, 1.16)	.31	.48
Birth <32weeks			Odds ratio (95% CI)		
Including twins	841	834	0.71 (0.39, 1.27)	.58	.30
Singletons only	833	818	0.79 (0.43, 1.44)	.38	.38
Time until birth			Hazard ratio (95% CI)		
Including twins	835	831	0.95 (0.91, 1.00)	.02	.14
Singletons only	827	815	0.96 (0.91, 1.01)	<.001	.11
*I^2^*			0^c^		

a In all studies, there are 1046 treated and 1043 control subjects. Some studies are missing one or more variables needed for analysis.

b Logistic model with random effect of study and terms for treatment, gestational age at randomisation, and preterm birth history for odds ratio; comparable Cox model for hazard ratio.

c *I^2^* is negative, which is conventionally reported as 0.

**TABLE 5:** Effect of treatment on measures of pregnancy duration (metronidazole trials), imputing individual participant data results of studies for which only aggregate data were available

Results	Treated (*n*)^a^	Controls (*n*)^a^	Unadjusted estimate of treatment effect^b^	Adjusted estimate of treatment effect^c^	*p*-value for interaction of treatment & gestational age at randomisation^d^	*p*-value for interaction of treatment and preterm birth history^e^
Birth <37weeks			Odds ratio (95% CI)	Odds ratio (95% CI)		
Including twins	2368	2284	0.98 (0.84, 1.14)	0.95 (0.81, 1.11)	.81	.63
Singletons only	2359	2276	0.99 (0.85, 1.15)	0.95 (0.81, 1.11)	.74	.61
*I^2^*				59%		
Birth <34weeks			Odds ratio (95% CI)	Odds ratio (95% CI)		
Including twins	2368	2286	0.91 (0.72, 1.16)	0.88 (0.69, 1.13)	.51	.69
Singletons only	2359	2278	0.92 (0.72, 1.17)	0.88 (0.68, 1.13)	.52	.69
Birth <32weeks			Odds ratio (95% CI)	Odds ratio (95% CI)		
Including twins	2369	2286	1.01 (0.73, 1.40)	1.01 (0.74, 1.37)	.59	.28
Singletons only	2360	2278	1.02 (0.74, 1.41)	1.01 (0.74, 1.38)	.61	.28
Time until birth			Hazard ratio (95% CI)	Hazard ratio (95% CI)		
Including twins	2367	2282	1.01 (0.98, 1.03)	1.00 (0.97, 1.02)	1.00	.99
Singletons only	2358	2274	1.01 (0.99, 1.03)	1.00 (0.97, 1.03)	1.00	.99
*I^2^*				74%		

a n’s are average number used in the analysis; different imputations can produce slightly different n’s.

b Logistic model with random effect of study for odds ratio; Cox model with random effect of study for hazard ratio.

c Logistic model with random effect of study and terms for treatment, gestational age at randomisation, and history of preterm birth (3-level) for odds ratio; comparable Cox model for hazard ratio.

d The p-value for the interaction of treatment and gestational age at randomisation has terms for treatment, gestational age at randomisation, history of preterm birth (3-levels), and the interaction of treatment and gestational age at randomisation.

e The p-value for the interaction of treatment and history of preterm birth has treatment, gestational age at randomisation, history of preterm birth (2-level), and the interaction of treatment and history of preterm birth.

**TABLE 6:** Effect of treatment on measures of pregnancy duration (clindamycin trials), imputing individual participant data results of studies for which only aggregate data were available

Results	Treated (*n*)^a^	Controls (*n*)^a^	Unadjusted estimate of treatment effect^b^	Adjusted estimate of treatment effect^c^	*p*-value for interaction of treatment and gestational age at randomisation^d^	*p*-value for interaction of treatment and preterm birth history^e^
Birth <37weeks			Odds ratio (95% CI)	Odds ratio (95% CI)		
Including twins	3500	2574	0.97 (0.80, 1.18)	0.90 (0.72, 1.12)	.13	.30
Singletons only	3458	2528	1.01 (0.84, 1.23)	0.94 (0.76, 1.15)	.04	.25
*I^2^*				0^f^		
Birth <34weeks			Odds ratio (95% CI)	Odds ratio (95% CI)		
Including twins	3500	2574	0.96 (0.69, 1.34)	0.85 (0.58, 1.24)	.17	.94
Singletons only	3458	2528	0.98 (0.66, 1.45)	0.86 (0.56, 1.32)	.15	.98
Birth <32weeks			Odds ratio (95% CI)	Odds ratio (95% CI)		
Including twins	3500	2574	0.99 (0.65, 1.50)	0.97 (0.60, 1.56)	.32	.59
Singletons only	3458	2528	1.07 (0.69, 1.67)	1.07 (0.65, 1.75)	.16	.70
Time until birth			Hazard ratio (95% CI)	Hazard ratio (95% CI)		
Including twins	3484	2571	1.00 (0.97, 1.02)	0.99 (0.97, 1.02)	.99	.17
Singletons only	3452	2525	1.00 (0.98, 1.02)	0.99 (0.97, 1.02)	.99	.16
*I^2^*				0^f^		

a The PREMEVA study used 2:1 randomisation and is primarily responsible for the difference in sample size between treatment and control in the combined studies. N’s are average number used in the analysis; different imputations can produce slightly different N’s.

b Logistic model with random effect of study for odds ratio; Cox model with random effect of study for hazard ratio.

c Logistic model with random effect of study and terms for treatment, gestational age at randomisation, and history of preterm birth for odds ratio; comparable Cox model for hazard ratio.

d The *p*-value for the interaction of treatment and gestational age at randomisation has terms for treatment, gestational age at randomisation, history of preterm birth (3-level), and the interaction between treatment and gestational age at randomisation.

e The *p*-value for the interaction of treatment and history of preterm birth has terms for treatment, gestational age at randomisation, history of preterm birth (two levels), and the interaction of treatment and history of preterm birth.

f *I^2^* is negative, which is conventionally reported as zero.

## Data Availability

The data for this study remain the property of the individual study investigators. Requests to access these data should be made directly to them.

## Synopsis

### Study questions

Does treatment of asymptomatic pregnant individuals for bacterial vaginosis (BV) prevent preterm delivery (PTD) or prolong pregnancy? Does the effect of treatment differ by gestation at randomisation, PTD history, or metronidazole versus clindamycin treatment?

### What is known

There have been numerous aggregate data meta-analyses, with inconsistent results, but aggregate data meta-analyses are less suitable to address heterogeneous treatment effects.

### What this study adds

This is the only study to pool individual participant data (IPD) for BV treatment and impute IPD of studies not providing IPD. Before the imputation of nonparticipating studies, clindamycin, not metronidazole, prolonged pregnancy, and reduced PTD. Following imputation of nonparticipating studies, treatment of BV did not prolong pregnancy or reduce PTD, regardless of antibiotic used, gestation at randomisation, or obstetric history.
