# Supplementary material for: Antibiotic treatment of bacterial vaginosis to prevent preterm delivery: Systematic review and individual participant data meta-analysis
Source: Paediatr Perinat Epidemiol. Author manuscript; Available in PMC 2023 May 10. (PMC10171232; doi:10.1111/ppe.12947)
Supplement: supplementary appendix [file NIHMS1892991-supplement-supplementary_appendix.pdf]

SUPPLEMENTAL APPENDIX: IMPUTATION METHODS

General Principles

Donor subjects were selected with replacement from studies for which we had IPD following constraints on the gestational age at randomization and whether multiple births were included in the study. For some studies, donors were selected with probabilities specified to produce distributions of additional variables consistent with reported information. This selection process is analogous to the use of sampling weights in surveys to adjust for unequal frequencies of response. For the variables used in sampling, the resulting imputed data sets were not statistically significantly different from published results. In general, the imputed data sets exactly matched the aggregate, reported information on number of term and PTBs by treatment arm.

Study-Specific Imputation Methods

Metronidazole studies

Morales randomized 80 women who had previous preterm births and current singleton pregnancies to metronidazole (n=44) or placebo (n=36). The women were between 13 and 20 weeks of gestational age at randomization. In the metronidazole group, 2 gave birth before 34 weeks, 6 others before 37 weeks, and 36 at term. In the placebo group, 4 gave birth before 34 weeks, 12 others before 37 weeks, and 20 at term. The imputed data sets have exactly the same number of very preterm, preterm and term births by treatment group.

McDonald randomized women with singleton pregnancies who were at least 17 years old to metronidazole (n=429) or placebo (n=428). They were screened around 19 weeks, but randomized around 24 weeks. Donors were selected who were randomized between 20 and 28 weeks. In the MZ group, 22 women with previous preterm births had two preterm births and 407 women without previous preterm births had 29 preterm births. In the control group, 24 women with previous preterm births had ten preterm births and 404 women without previous preterm births had 22 preterm births. The imputed data sets have exactly the same number of preterm and term births by treatment group and previous preterm status.

Moniri randomized 120 women to metronidazole (n=60) or control (n=60). Randomization occurred between 20 and 34 weeks of gestational age. In both groups there were 2 premature births. The imputed data sets have exactly the same number of preterm and term births by treatment group.

Clindamycin studies

Subtil et al randomized 1904 women to clindamycin and 956 to placebo, at less than 15 weeks' gestation. Four of the five studies with data using clindamycin randomized women at this early gestational range. The Kiss study was not used to create imputation donors because gestational age at delivery was not provided in individual weeks. An imputed data set was created by randomly selecting individuals from the four comparable studies so that counts

match published treatment and outcome results. Five separate data sets were created to allow for multiple imputation inference (Rubin 1987). The summary characteristics of the imputed data sets exactly match the known summaries of the actual data.

Joesoef et al (1995) provides counts of number of subjects receiving clindamycin (n=340) and placebo (n=341), number very preterm (less than 32 weeks), number preterm (less than 37 weeks), and a fact about randomization (14-26 weeks). Information on rates of low birth weights (of current pregnancies) and mother's age (in ranges) also are available. An imputed data set was created by randomly selecting individuals from the four comparable studies so that counts match published treatment and outcome results. Sampling weights analogous to post stratification sampling weights were created to give donors relative frequencies to match the mother's age distribution. Five data sets were created. The summary characteristics of the imputed data sets exactly match the known summaries of the actual data or are not statistically significantly different in terms of low birth weight and mother's age categories.

Vermeulen and Bruinse (1999) included twenty-two women with BV, eleven each in clindamycin and placebo groups. One in each group had a delivery before 34 weeks. Randomization occurred before 26 weeks of gestation. Women had singleton pregnancies. An imputed data set was created by randomly selecting individuals from the four comparable studies so that counts match published treatment and outcome results.

Kekki (2001) provides counts of subjects receiving clindamycin (n=187) and placebo (n=188), number of preterm (less than 37 weeks), a fact about randomization (12-19 weeks), and age ranges of mothers. An imputed data set was created by randomly selecting individuals from the four comparable studies so that counts match published treatment and outcome results. All mothers had singleton pregnancies. Sampling weights based on empirical likelihood methods were created to give donors relative frequencies to resemble the reported mean mother's age in both groups. Five data sets were created. The summary characteristics of the imputed data sets exactly match the known summaries of the actual data. The average mother's age in the imputed data sets is not statistically different from the reported mother's age in either the treatment or placebo groups.

Guaschino (2003) randomized 112 women to clindamycin or no treatment; 55 to clindamycin and 57 to control. Six in each group were lost to follow up. The study provides counts of number of preterm (less than 37 weeks), a fact about randomization (14-25 weeks), and some other information, including the average gestational age at randomization by group. An imputed data set was created by randomly selecting individuals from the four comparable studies so that counts match published treatment and outcome results. All mothers had singleton pregnancies. Sampling weights based on empirical likelihood methods were created to give donors relative frequencies to resemble the reported mean gestational age at randomization in both groups. The imputed data sets have exactly the same number of preterm

and term births by treatment group. The average gestational age at randomization is not statistically different from that reported in either the treatment groups.

Giuffrida and Mangiacasale (2006) randomized sixty BV positive women to either clindamycin (n=30) or hydrogen peroxide (n=30). The women were at 13-16 weeks of gestation at the time of randomization. There were no preterm birth events. An imputed data set was created by randomly selecting individuals from the four comparable studies so that counts match published treatment and outcome results.

Kiss randomized women to treatment with clindamycin (n=188) or control (n=187). The exact gestational age at delivery was not available, but ranges were reported as less than 33 weeks, 33 to 36 weeks, and 37 weeks or more. Women were randomized at either 16 or 21 weeks, with each value likely representing a range of gestational ages at randomization. For each woman in the Kiss database, donors were selected from other studies using CM by matching on twin status, previous preterm status, nulliparous status, gestational age at randomization within two-and-a-half weeks, and range of gestational age at delivery. Indicators of extremely early, very early, and premature birth were updated when needed to be consistent with the imputed gestational age at delivery.
