# Supplementary material for: Antibiotic treatment of bacterial vaginosis to prevent preterm delivery: Systematic review and individual participant data meta-analysis
Source: Paediatr Perinat Epidemiol. Author manuscript; Available in PMC 2023 May 10. (PMC10171232; doi:10.1111/ppe.12947)

Supplemental Figure 1: Metronidazole versus Control, Stratified by Gestational Age at Randomization

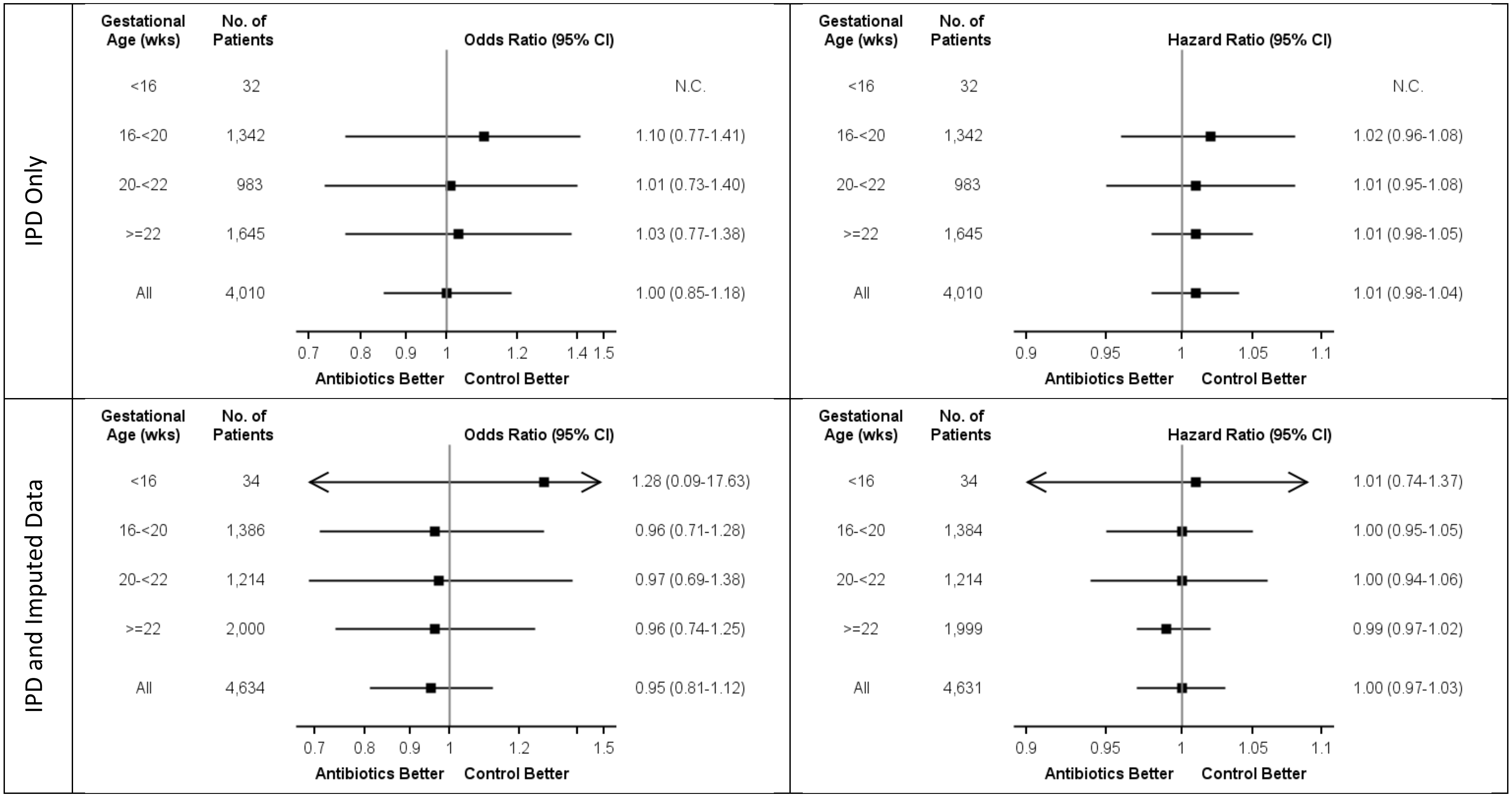

Supplemental Figure 2: Metronidazole versus Control, Stratified by Prior Preterm Birth

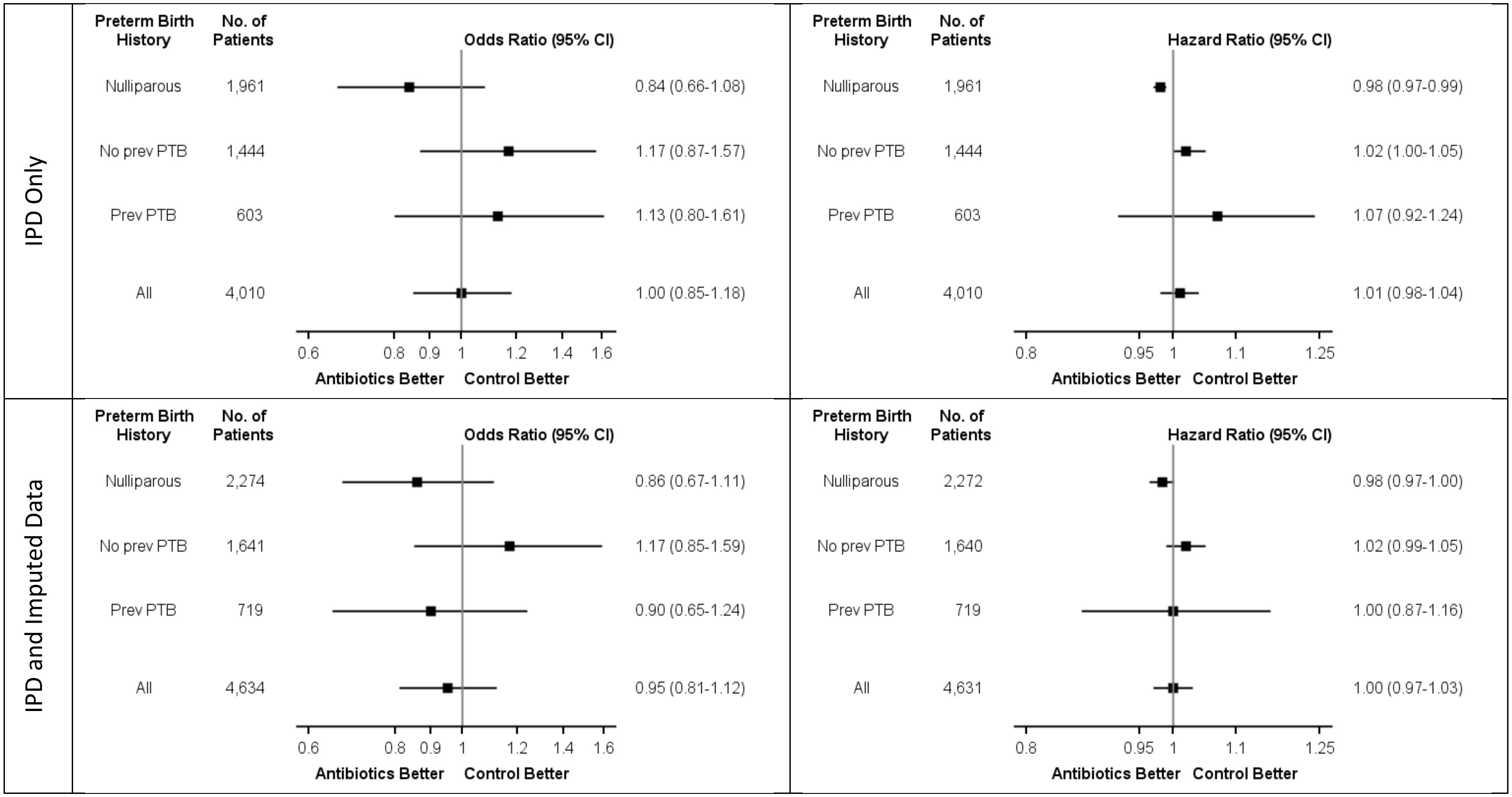

Supplemental Figure 3: Clindamycin versus Control, Stratified by Gestational Age at Randomization

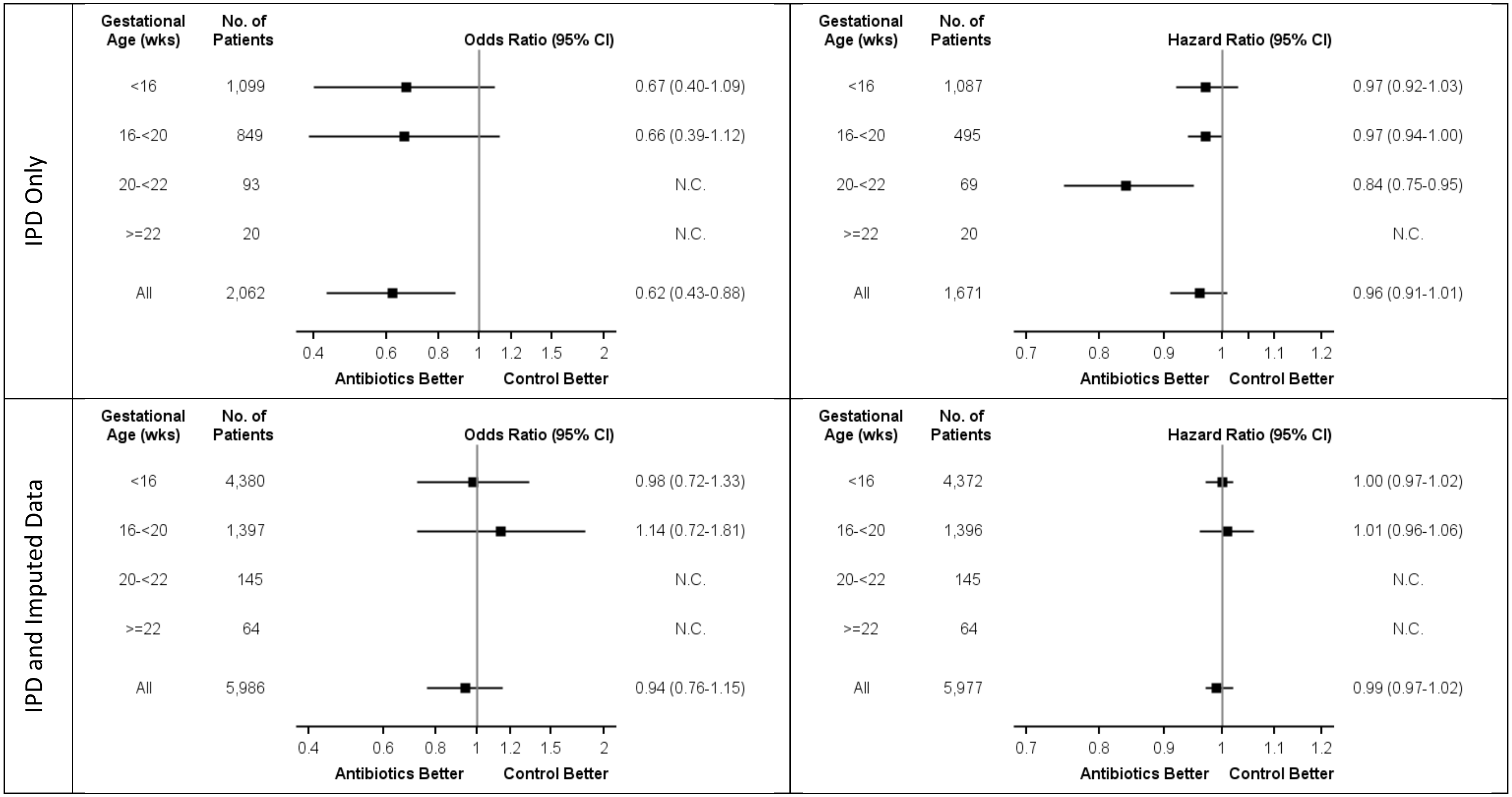

Supplement: supplementary figures [file NIHMS1892991-supplement-supplementary_figures.pdf]
