# Supplementary material for: Antibiotic treatment of bacterial vaginosis to prevent preterm delivery: Systematic review and individual participant data meta-analysis
Source: Paediatr Perinat Epidemiol. Author manuscript; Available in PMC 2023 May 10. (PMC10171232; doi:10.1111/ppe.12947)
Supplement: supplementary tables [file NIHMS1892991-supplement-supplementary_tables.pdf]

Table S1: Characteristics of Studies Providing Individual Patient Data

Study	N*	N used†	Active treatment‡	Control treatment	Allocation ratio	BV definition	Gestation at randomization	Sonography for gestational age
Hauth, 1995	263	256	MZ, 250 mg tid x 7; EM 333 mg/d x 14, repeat if positive at 2-4 wks	Matching placebos, repeated if still positive at 2-4 wks	2:1, variable block, computer generated	¾ Amsel criteria, Nugent score 7+	22-24 weeks	Yes
NICHD BV, 2000	1944	1910 OR; 1908 HR	MZ, 2 gram x 2, on 2 occasions regardless of positivity	Matching placebo	1:1, urn, stratified by study site	Nugent score 7+ and pH>4.4	16-23 weeks	Yes, before randomization
NICHD TV, 2001	238	233	As NICHD BV	As NICHD BV	As NICHD BV	As NICHD BV	As NICHD BV	As NICHD BV
Odendaal, 2002	277	276	MZ, 400 mg bid x 2 days repeated at 4 weeks if still positive	Vitamin C, 100 mg bid x 2 days repeated at 4 weeks if still positive	1:1, balanced block design	¾ Amsel or Lacto <2+ (Spiegel)	15-26 weeks	Yes if <24 weeks, no otherwise
Ugwumadu, 2003	410	391	Oral CM, 300 mg bid x 5,	Matching placebo	1:1, simple randomizatio n	Nugent score 7+	12-21 weeks	Yes (could be done after randomization)
Lamont, 2003	417<br>412§	OR 407<br>402§<br><br>HR 398<br>393§	Topical CM, 3 evenings	CM vehicle, 3 evenings	1:1, random block	Nugent score 7+, study also included women with	13-20 weeks	Yes, before randomization

						scores 4-6§		
NICHD fFN, 2003	190	185 OR; 184 HR	MZ 250 mg tid, EM 333 mg 4/d x 10 days	Matching placebos	1:1 urn, stratified by study site	Nugent score 7+	21-25 weeks	Yes, before randomization
Kiss, 2004	375 372§	359 356 OR only	Topical CM, 6 days; oral CM 200 bid x 7 if positive at 24-27 wks (topical MZ if positive for TV	None, all women screened, and then randomized to receive screening results	1:1, computer generated	Nugent score 7+	15-19 weeks	Yes (could be done any time up to 19 wks)
Shennan, 2006	13	11	MZ, 400 mg tid x 7 d	Matching placebo	1:1, stratified by week of positive fFn and clinical site	Nugent score 7+	24-27 weeks	Yes
Larsson, 2006	819 800§	809 790§	Topical CM, 7 evenings, repeated if positive at 24 or 31 weeks	None (Zelen design)	1:1, block size 10	Nugent score 6+, changed to Hay-Ison score	10-14 weeks	Best clinical estimate from Swedish Medical Birth Registry
Goldenberg, 2006	1170||	1101 1085¶	MZ, EM 250 mg tid x 7d	Matching placebo	1:1, permuted block, stratified by clinical site	Nugent score 7+	20-24 weeks	No
Gupta, 2013	800	0	Topical CM 100 mg; clotrimazole	None	1:1 computer generated	Nugent score 7+	12-24 weeks	If available; number with

			100 mg, 7 evenings					sonogram unknown
Hoffman, 2018	68	68	CM, 300 mg bid x 5 d	Matching placebo	1:1, blocks of 6	Nugent score 7+ and pH 5+	13-20 weeks	If available; sonography not routine

* Number of observations in received data file

† Number included in analysis, typically excluding women with missing outcome, gestation at randomization and/or obstetrical history. The duration of pregnancy is used for the hazard ratio (HR), whereas an indication of birth before 37 weeks is used for the odds ratio (OR); the number for each analysis is indicated if they differ.

‡ MZ=metronidazole; CM=clindamycin; EM=erythromycin

§ Data provided do not allow women with abnormal flora and BV to be differentiated

|| Estimated number of women with BV based on published paper. Received data excluded women who were missing outcome data.

¶ Number after excluding twin pregnancies

Table S2: Characteristics of Studies Not Providing Individual Patient Data

Study	N*	N†	N (%) preterm‡	Active treatment§	Control treatment	Allocation ratio	BV definition	Gestation at randomization	Sonography for gestational age
Duff, 1991	108	0‖	Not stated	AX, 500 mg tid x 14 d	Matching placebos	Adaptive randomization, biased coin; blocks of 6	Nugent score 7+ and Amsel	15-25 weeks	Not stated
Morales, 1994	94	80	24 (30%)	MZ, 250 mg tid x 7d	Identical appearing Vitamin C tablet	1:1	Amsel criteria	13-20 weeks	Yes, before randomization
Joesoef, 1995	745	681	97 (14%)	Topical CM, 7 evenings	Matching placebo	1:1, permuted blocks of 6, stratified by study site	Nugent score 7+ and pH>4.5	14-26 weeks	Yes, before randomization
McDonald 1997	490	480	34 (7%)	MZ, 400 mg bid x 2d, repeated in 4 weeks if BV present	Matching placebo	1:1, blocks of 16 stratified by study site	Spiegel Gram stain score	16-26 weeks	Yes, <18 weeks
Vermeulen, 1999	22	22	2 (9%), <34 weeks	Topical CM, 7 evenings at 26 and 32 weeks	Matching placebo	1:1, blocks of 4, stratified by BV status and study site	Nugent score 7+	<26 weeks	Yes, timing not stated
Kekki, 2001	375	375	16 (4%)	Topical CM every evening x 7d	Matching placebo	1:1, “block randomization within each center”	Spiegel Gram stain score	10-17 weeks	Yes, timing not stated

Guaschino, 2003	112	100	14 (14%)	Topical CM, 7 evenings	No treatment	1:1, stratified by study site	Nugent score 7+	13-20 weeks	Yes, before randomization
Giuffrida, 2006	60	60	Mean 38.2 weeks	Topical CM ovules, 100 mg x 3d, repeat at 3 weeks if still positive	Hydrogen peroxide cream, 0.5% x 5 d, repeated at 3 weeks if still positive	1:1	Unclear	13-16 weeks	Yes, before randomization
Moniri, 2009	120	120	4 (3%)	MZ, 500 mg bid x 7d	Untreated, uninformed of BV	Not stated	Amsel criteria	20-34 weeks	Not stated
Subtil, 2018	2869	2860	200 (7%)	Oral CM, bid x 4 days for 1 or 3 courses 1 month apart	Matching placebo 2, 0 or 3 courses	1:1:1, blocks of 6, stratified by study site	Nugent score 7+	<15 weeks	Best obstetric estimate

* Number of pregnancies in published paper.

† Number of pregnancies in published paper with BV and outcome data.

‡ Among women with outcome data present in published paper.

§ AX=amoxicillin; MZ=metronidazole; CM=clindamycin.

|| Published results state only that gestational age at birth did not differ significantly between treated and control groups.

Table S3: Risk of bias of studies providing individual data

	Random sequence generation	Allocation concealment	Blinding of participants and personnel	Blinding outcome assessment	Incomplete outcome data	Selective reporting	Other
Hauth, 1995	+	+	+	+	+	?[*]	+
NICHD BV, 2000	+	+	+	+	+	+[†]	+
NICHD TV, 2001	+	+	+	+	+	+[†]	+
Odendaal, 2002	+	-	+	+	+	?[*]	+
Lamont, 2003	+	+	+	+	+	?[*]	+
NICHD fFn, 2003	+	+	+	+	+	+	+[‡]
Ugwumadu, 2003	+	+	+	+	+	?	+[§]
Kiss, 2004	+	+	-[‖]	?[‖]	+	?[*]	+
Goldenberg, 2006	+	+	+	+	+[¶]	+	+
Larsson, 2006	+	+	-[‖]	?[‖]	?[#]	?[*]	?[**]
Shennan, 2006	+	+	+	+	+	+	?[††]
Gupta, 2013	?	?	?	?	-[‡‡]	?[*]	?[a]
Hoffman, 2018	+	+	+	+	+	+	+

+ = low risk of bias; - = high risk of bias; ? = unclear risk of bias

* Study protocol unavailable, therefore impossible to compare reported and planned outcomes.

† All primary and secondary outcomes in protocol were reported. Additional outcomes and subgroup analysis also reported. All were negative.

‡ Baseline difference in history of preterm birth differs between treatment and control groups in the entire study population, but not among the subset of BV+ women that are included in this meta-analysis.

§ Primary outcome in protocol agrees with outcome in published report. Protocol lists no secondary outcomes or planned subgroup analyses.

|| Some degree of unblinding is inevitable under the Zelen design employed. Since patients randomized to active treatment, and their managing clinicians, were aware of BV-positivity, clinical management (such as decision to deliver for a complication) might impact gestational age in these women. The degree to which this happened is speculative but we judge it to be limited.

¶ Pregnancy outcome data unavailable for 5.9% of BV+ women randomized to antibiotic and 7.3% of those randomized to placebo. Reasons for withdrawal did not differ substantially by treatment group.

Small number of post-randomization exclusions not due to missing outcomes (twins, treatment outside of study, indicated preterm birth)

** Target sample size not reached, no explanation provided.

†† Target sample size not reached, study terminated due to futility.

‡‡ Women who developed a pregnancy complication after randomization were excluded. Number of such women not provided.

a. Sample size calculation unclear.

Table S4: Risk of Bias of Studies Not Providing Individual Patient Data

	Random sequence generation	Allocation concealment	Blinding of participants and personnel	Blinding outcome assessment	Incomplete outcome data	Selective reporting	Other bias
Duff, 1991	+	?*	+	+	?	?†	?
Morales, 1994	+	?*	+	+	-‡	?†	?
Joesoef, 1995	+	+	+	+	?	?†	+
McDonald, 1997	+	?*	+	+	+	?†	?§
Vermeulen, 1999	?	?	+	+	+	?†	+
Kekki, 2001	?	+	+	+	+	?†	+
Guaschino, 2003	?	+	-	-	+	?	?||
Giuffrida, 2006	?	?	-	-	+	?†	+
Moniri, 2009	?	?	-	-	+	-¶	+
Subtil, 2018	+	+	+	+	+	+	+

+ = low risk of bias; - = high risk of bias; ? = unclear risk of bias

* Not stated whether the individual preparing the randomization sequence had any contact with trial participants.

† Study protocol unavailable; therefore impossible to determine whether reported outcomes agree with those stated in the protocol.

‡ Post-randomization exclusion of women who did not receive assigned treatment.

§ Study terminated early due to futility.

|| Substantial, although not statistically significant, difference in baseline characteristics.

¶ Three outcomes noted in abstract not reported in results.

** Publication was part of a "larger multi-center double blind, randomized placebo-controlled trial of the efficacy of 2% clindamycin cream for the treatment of bacterial vaginosis in the prevention of prematurity"; 13/142 women lost to follow up, which is higher than most trials

Table S5: Estimated odds ratio of treatment on preterm birth, for each study providing IPD. Odds ratios <1 indicate that treatment reduces preterm birth.

	N	Non-missing N	Odds Ratio (95% Confidence Limits)*	P-value for interaction of treatment * gestation at randomization	P-value for interaction of treatment * obstetric history
Metronidazole Studies					
Hauth'95	263	256	0.45 (0.25-0.78)	0.49	0.52
Carey'00 BV	1944	1910	0.96 (0.72-1.27)	0.17	0.29
Klebanoff'01 TV	238	233	1.85 (0.91-3.93)	0.17	0.06
Odendaal'02	277	276	1.64 (0.94-2.90)	0.99	0.18
Andrews'03 fFn	190	185	0.70 (0.29-1.65)	0.31	0.27
Shennan'06†	13	11	2.67 (0.15-3.40)	--	--
Goldenberg'06	1101	1101	1.02 (0.77-1.34)	0.99	0.92
Singletons only	1085	1085	1.03 (0.78-1.37)	0.84	0.95
Clindamycin Studies					
Ugwumadu'03	410	391	0.47 (0.24-0.87)	0.88	0.41
Lamont'03	417	407	0.61 (0.29-1.24)	0.97	0.73
No twins	412	402	0.68 (0.32-1.42)	0.58	0.75
Kiss'04	375	359	0.55 (0.22-1.28)	1.00	1.00
No twins	372	356	0.57 (0.22-1.40)	1.00	1.00
Larsson'06	819	809	0.90 (0.48-1.68)	0.61	0.98
No twins	800	790	1.10 (0.54-2.27)	0.53	0.83
Hoffman'18‡	68	68	-	-	-

* Odds ratios are also adjusted for gestational age at randomization and history of preterm birth as a 3-level variable

† Due to small sample size, the individual results use only treatment as a predictor variable. The study is included in overall results with all variables.

‡ There were no preterm births in the treatment arm, but some in the placebo arm. As a result, the odds ratio is estimated to be zero; usual hypothesis testing results are not reported because estimate is on the edge of the parameter space. The study is included in overall results with all subjects.

Table S6: Estimated hazard ratio of treatment on gestational age at birth, capped at 37 weeks, for studies providing IPD. Hazard ratios <1 indicate that treatment prolongs pregnancy.

Hazard Ratio	N	N used	Hazard Ratio (95% Confidence Limits)*		P-value for interaction treatment * gestation at randomizatio n	P-value for interaction treatment * obstetric history
			Metronidazole Studies			
Hauth’95	263	256	0.80 (0.61-1.04)		0.54	0.90
Carey’00 BV	1944	1908	0.99 (0.91-1.09)		0.69	0.60
Klebanoff’01 TV	238	233	1.12 (0.86-1.45)		0.73	0.10
Odendaal’02	277	276	1.13 (0.89-1.43)		0.83	0.31
Andrews’03 fFn	190	184	0.94 (0.70-1.25)		0.74	0.32
Shennan’06	13	11	3.51 (0.71-17.31)		0.75	0.99
Goldenberg’06	1101	1101	1.00 (0.89-1.13)		0.97	0.97
Singletons only	1085	1085	1.01 (0.89-1.13)		0.96	0.98
			Clindamycin Studies			
Ugwumadu’03	410	391	0.91 (0.74-1.11)		0.96	0.23
Lamont’03	417	398	0.95 (0.78-1.16)		0.92	0.96
Singletons only	412	393	0.96 (0.79-1.17)		0.92	0.95
Kiss’04†	375	-	-	-	-	-
Singletons only	372	-	-	-	-	-
Larsson’06	819	809	0.99 (0.87-1.14)		0.91	0.99
Singletons only	800	790	1.00 (0.87-1.15)		0.92	0.98
Hoffman’18	68	68	0.75 (0.46-1.22)		0.73	‡

* Hazard ratios are also adjusted for gestational age at randomization and history of preterm birth as a 3-level variable

† Gestational age provided as a categorical variable only. Therefore exact gestation at delivery could not be ascertained.

‡ Due to small sample size and arrangement of subjects across risk and treatment categories, it is not possible to calculate the interaction

Table S7: Secondary Outcomes, Not Reported Elsewhere

Outcome	Treated (n)	Controls (n)	Estimate of treatment effect*
Stillbirth or miscarriage			Odds ratio (95% CI)
Twins included	3033	2955	0.81 (0.55, 1.19)
Singletons only	3015	2930	0.82 (0.56, 1.22)
Stillbirth, miscarriage, or neonatal death			Odds ratio (95% CI)
Twins included	1914†	1844	0.95 (063, 1.44)
Singletons only	1905	1837	0.98 (0.65, 1.48)
Birth weight‡ <2500 g			Odds ratio (95% CI)
Twins included	2975	2894	0.88 (0.75, 1.04)
Singletons only	2958	2872	0.89 (0.76, 1.05)
Birth weight‡ <1500 g			Odds ratio (95% CI)
Twins included	2975	2894	1.05 (0.76, 1.45)
Singletons only	2958	2872	1.05 (0.76, 1.46)

*Models are adjusted for study, gestational age at randomization, and history of preterm birth.

†Few studies had an indication of neonatal death, so the number of subjects missing the composite of stillbirth, miscarriage, or neonatal death is larger than the number of subjects missing still birth or miscarriage.

‡ Not a pre-specified secondary outcome

Table S8: Estimated odds ratio* of treatment on preterm birth, by metronidazole or clindamycin treatment, including imputed results from studies without IPD. Odds ratios <1 indicate that treatment reduces preterm birth. Results are presented overall and excluding each individual study.

	n*	Treated (n)	Controls (n)*	Odds Ratio (95% Confidence Limits)†	P-value for interaction of treatment * gestation at randomization	P-value for interaction of treatment * obstetric history
Overall	4652	2368	2284	0.95 (0.81-1.11)	0.81	0.63
Singletons only	4634	2359	2276	0.95 (0.81-1.12)	0.74	0.61
Metronidazole						
Exclude Hauth	4378	2188	2191	1.02 (0.86-1.21)	0.43	0.76
Exclude Carey	2724	1411	1314	0.94 (0.78-1.15)	0.64	0.20
Exclude Klebanoff	4401	2231	2171	0.92 (0.78-1.08)	0.64	0.34
Exclude Odendaal	4358	2219	2140	0.91 (0.77-1.07)	0.55	0.22
Exclude Andrews	4449	2273	2177	0.97 (0.82-1.14)	0.57	0.85
Exclude Shennan	4623	2353	2271	0.95 (0.81-1.11)	0.82	0.53
Exclude Goldenberg	3549	1825	1725	0.91 (0.75-1.10)	0.90	0.67
Exclude Morales	4554	2315	2240	0.99 (0.84-1.16)	0.95	0.83
Exclude McDonald	4155	2117	2039	0.96 (0.82-1.13)	0.65	0.94
Exclude Moniri	4515	2299	2216	0.95 (0.81-1.12)	0.73	0.60
Overall	6075	3500	2574	0.90 (0.72-1.12)	0.13	0.30
Singletons only	5986	3458	2528	0.94 (0.76-1.15)	0.04	0.25
Clindamycin						
Exclude Ugwumadu	5595	3260	2335	1.02 (0.82-1.27)	0.05	0.50
Exclude Lamont	5584	3256	2328	0.96 (0.77-1.19)	0.05	0.18
Exclude Kiss	5614	3271	2343	0.93 (0.75-1.14)	0.04	0.21
Exclude Larsson	5196	3060	2136	0.92 (0.74-1.15)	0.06	0.26
Exclude Hoffman	5918	3423	2495	0.98 (0.79-1.21)	0.06	0.20
Exclude Subtil	3238	1626	1612	0.80 (0.60-1.06)	0.18	0.38
Exclude Joesoef	5318	3123	2195	0.90 (0.70-1.14)	0.01	0.25
Exclude Vermeulen	5965	3448	2517	0.94 (0.76-1.16)	0.04	0.25
Exclude Kekki	5619	3275	2344	0.93 (0.75-1.15)	0.03	0.25
Exclude Gauschino	5886	3409	2477	0.96 (0.78-1.19)	0.14	0.46
Exclude Giuffrida	5927	3429	2498	0.94 (0.76-1.15)	0.04	0.26

*N's are average number used in analysis; different imputations can produce slightly different N's. Rounding can cause number overall to be different than sum of number in treatment and control. Twins are not included except in the overall results

†Model includes terms for gestational age at randomization and history of preterm birth (as a 3-level variable). Twin pregnancies are included only in the overall results.

Table S9: Estimated hazard ratio* of treatment on gestational age at birth, capped at 37 weeks, by metronidazole or clindamycin treatment, including imputed results from studies without IPD. Hazard ratios <1 indicate that treatment prolongs pregnancy. Results are presented overall and excluding each individual study.

	n*	Treated (n)*	Controls (n)*	Hazard Ratio (95% Confidence Limits)†	P-value for interaction of treatment * gestation at randomization	P-value for interaction of treatment * obstetric history
Overall	4649	2367	2282	0.99 (0.96-1.03)	1.00	0.99
Singletons only	4631	2357	2274	1.00 (0.97-1.03)	1.00	0.99
Metronidazole						
Exclude Hauth	4376	2187	2189	1.00 (0.98-1.03)	0.99	0.73
Exclude Carey	2724	1411	1313	0.99 (0.94-1.04)	1.00	0.61
Exclude Klebanoff	4398	2230	2169	0.99 (0.96-1.02)	1.00	0.75
Exclude Odendaal	4355	2218	2138	0.99 (0.96-1.02)	1.00	0.56
Exclude Andrews	4447	2272	2176	1.00 (0.97-1.03)	1.00	0.81
Exclude Shennan	4620	2352	2269	1.00 (0.97-1.02)	1.00	0.95
Exclude Goldenberg	3536	1824	1723	1.00 (0.96-1.03)	1.00	0.97
Exclude Morales	4551	2314	2238	1.00 (0.98-1.03)	1.00	0.56
Exclude McDonald	4152	2116	2037	1.00 (0.97-1.03)	1.00	0.83
Exclude Moniri	4512	2298	2214	1.00 (0.97-1.03)	1.00	0.98
Overall	6066	3494	2571	0.99 (0.97-1.02)	0.99	0.17
Singletons only	5977	3452	2525	0.99 (0.97-1.02)	0.99	0.16
Clindamycin						
Exclude Ugwumadu	5586	3254	2332	1.00 (0.98-1.02)	0.99	0.41
Exclude Lamont	5584	3256	2328	1.00 (0.97-1.02)	0.99	0.16
Exclude Kiss	5605	3265	2340	0.99 (0.96-1.02)	0.99	0.09
Exclude Larsson	5187	3054	2133	0.99 (0.96-1.02)	0.99	0.14
Exclude Hoffman	5909	3417	2492	1.00 (0.97-1.02)	0.99	0.15
Exclude Subtil	3229	1609	1620	0.98 (0.95-1.01)	0.99	0.21
Exclude Joesoef	5309	3117	2192	0.99 (0.96-1.02)	0.99	0.20
Exclude Vermeulen	5956	3442	2514	0.99 (0.97-1.02)	0.99	0.16
Exclude Kekki	5610	3269	2341	0.99 (0.97-1.02)	0.99	0.15
Exclude Gauschino	5877	3403	2474	1.00 (0.97-1.02)	1.00	0.27
Exclude Giuffrida	5918	3423	2495	0.99 (0.97-1.02)	0.99	0.16

*N's are average number used in analysis; different imputations can produce slightly different N's. Rounding can cause number overall to be different than sum of number in treatment and control. Twins are not included except in the overall results

†Model includes terms for gestational age at randomization and history of preterm birth (as a 3-level variable). Twin pregnancies are included only in the overall results.
