# Supplementary material for: Antibiotic treatment of bacterial vaginosis to prevent preterm delivery: Systematic review and individual participant data meta-analysis
Source: Paediatr Perinat Epidemiol. Author manuscript; Available in PMC 2023 May 10. (PMC10171232; doi:10.1111/ppe.12947)
Supplement: supplementary figure s4 [file NIHMS1892991-supplement-supplementary_figure_s4.docx]

Supplemental Figure 4: Clindamycin versus Control, Stratified by Prior Preterm Birth

| IPD Only | 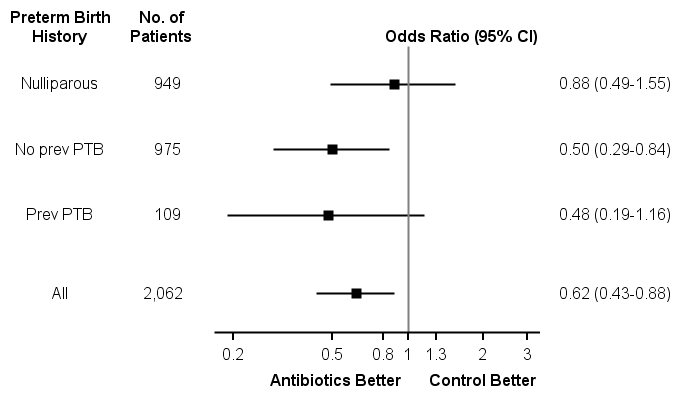 | 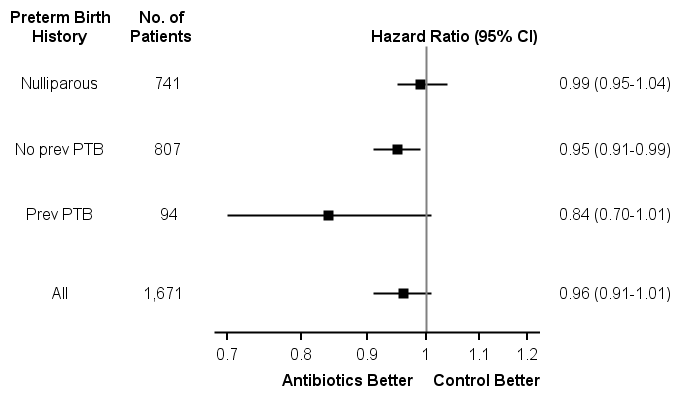 |
| --- | --- | --- |
| IPD and Imputed Data | 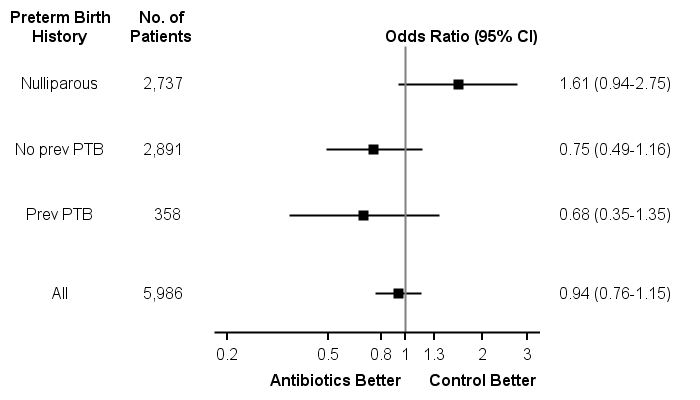 | 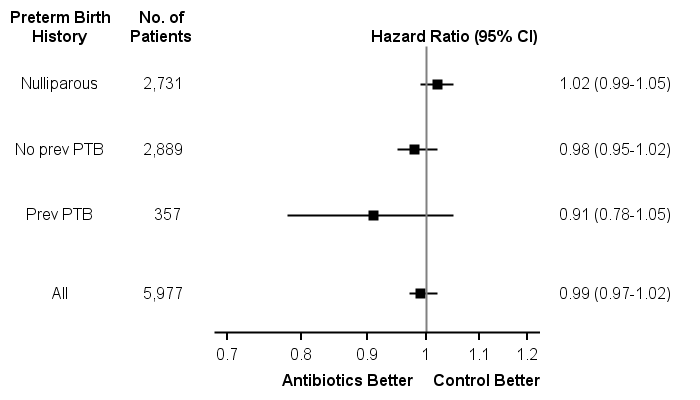 |
